# “I don’t know whose mouth has been on this”: youth nicotine and cannabis vaping practices in the context of the COVID-19 pandemic

**DOI:** 10.1186/s12889-022-14201-w

**Published:** 2022-09-23

**Authors:** Sabrina Islam, Kirsten Thompson, Melissa Abadi, Kristina Wharton, Sharon Lipperman-Kreda

**Affiliations:** 1 School of Public Health, University of California, Berkeley, 2121 Berkeley Way, Berkeley, CA 94704 USA; 2 Prevention Research Center, Pacific Institute for Research and Evaluation, 2030 Addison Street, Suite 410, CA 94704 Berkeley, USA; 3 Louisville Center, Pacific Institute for Research and Evaluation, 401 West Main Street, Suite 2100, KY 40202 Louisville, USA

**Keywords:** Vaping; nicotine; cannabis; youth; qualitative research

## Abstract

Early COVID-19 safety protocols urged physical and social distancing, resulting in minimal contact with others. As social contexts are central to vaping among youth, we used semi-structured interviews to describe how youth who vape are making sense of their use practices and adaptations. The qualitative analyses revealed changes in vaping frequency and access, social isolation shaping substance- and product-specific use, and motivations and outcomes of dual use of nicotine and cannabis which were closely linked to the pandemic. The findings indicate variation of youth vaping experiences within the early stages of the pandemic that should be attended to in future studies.

## Background

Public health safety measures adopted to control the spread of the coronavirus disease (COVID-19) imposed profound disruptions to everyday life including widespread closures of non-essential services, schools, restaurants, and recreational outlets (e.g., public parks, gyms) [1]. Social distancing, reduced interactions with others, and the unknowns of the severity of the COVID-19 pandemic also elevated fears and concerns related to youth mental and behavioral health, including how the pandemic might impact youth substance use [2, 3]. Particularly, targeted attention to vaping behaviors among youth emerged as this predominantly respiratory infection [4] presented as a major health event. Furthermore, subsequent diagnoses and symptoms were observed in people exclusively using ENDS (electronic nicotine delivery systems) or using both ENDS and combustible cigarettes [5, 6]. Comparable vulnerability to the COVID-19 infection from cannabis-vaping have been raised by clinicians as well [7]. Given these pronounced and continued health risks and the simultaneous proliferation of vaping nicotine and cannabis, it is necessary to better understand the vaping practices and motivations among youth during the COVID-19 pandemic.

A timely, mounting body of literature has examined the prevalence and nature of vaping during the early stages of the pandemic. Nationally, more than one-quarter of 12^th^ graders in the US vaped nicotine and nearly one-fifth vaped cannabis in the first year of the pandemic [8, 9]. A study focusing on younger people aged 13 to 24 reported that, among those reporting ever e-cigarette use, nicotine vaping behavior in the initial phase of the pandemic changed in terms of frequency, product type, and access [10]. Findings from a national survey also showed that perceived barriers to access and availability of nicotine and cannabis vaping products contributed to changes of use patterns but did not decrease use [11]. Overall, this research demonstrates that vaping practices since the onset of the COVID-19 pandemic involved changes for youth, especially as their use contexts were rapidly shifting. Less is known, however, about the ways in which nicotine and cannabis vaping contexts were altered.

A small but growing evidence base of qualitative research has documented some unique behavioral modifications stemming from the pandemic. For example, young adults who use nicotine identified an avoidance of sharing nicotine products and ENDS with others, buying nicotine products in bulk, and greater levels of nicotine use as specific changes attributed to the pandemic [12]. Notably, a study of young adults who regularly vape cannabis found that perceived risks of contracting COVID-19 did not correspond to vaping less cannabis or less nicotine [13]. Although this limited literature helps shed light on nicotine and cannabis vaping practices early in the pandemic (i.e., 2020), a gap exists in our understanding of what motivations, perceptions, and contexts are driving these behaviors. In light of the persistent popularity of vaping despite peaking health concerns and the magnitude of the COVID-19 pandemic, a mechanistic understanding of youth vaping experiences – inclusive of nicotine and cannabis products – is needed to drive comprehensive prevention and policy approaches forward. Therefore, the purpose of the present study is to explore how implications of COVID-19 (social distancing, closed businesses, home quarantine) influenced youth vaping in terms of access, opportunity, frequency, type of vaping, and dual use practices with other tobacco products and cannabis. Perceptions of how harm from vaping has been affected by COVID-19 is also explored.

In the current study, we conducted individual telephone interviews with youth to gain a richer understanding of contextual factors shaping nicotine and cannabis vaping during the critical first year of the pandemic. To develop the study framework and guide interpretation of findings, we drew from the socio-ecological framework that suggests vaping behaviors arise out of individual and environmental risk factors interacting [14, 15]. We also utilize the sense-making theory that suggests changes on a socio-ecological scale, such as that from a pandemic, can be collectively experienced yet trigger uncertainty and confusion that compels individuals to construct new meanings and understandings [16, 17]. Together, these approaches, situated within the continuously evolving social contexts associated with COVID-19, will enrich our understanding of youth nicotine and cannabis use behaviors to begin addressing limitations of the extant research.

## Methods

### Study design

Interview data were collected as part of a follow-up study to a project on youth vaping. The original 2018 study (1R03DA041899) used ecological momentary assessment (EMA) methods to examine daily exclusive and dual use of ENDS and tobacco cigarettes among youth. A follow-up study, consisting of an initial survey, two-week EMA, and in-depth interviewers, was conducted as an opportunity to enhance understanding of youth perceptions and practices of their vaping behaviors and other substance use, related health beliefs, and impacts from COVID-19.

Mandates on sheltering in place were enacted by the state of Kentucky on March 26, 2020, subsequent to the national state of emergency declared 13 days prior. Data collection took place in August 2020, which is referred to as the early stages of the pandemic in this paper. At the time of data collection, Kentucky residents were urged to stay at home, wear masks, and observe social distancing. In addition, non-essential industries, such as restaurants and gyms, and public schools and colleges were ordered to close.

### Study participants and sampling

We recruited the 50 youth who participated in the prior R03 study. At the time of the 2018 study, youth were between 13–17 years of age, lived within 100 miles of Louisville, KY, and reported past two-week nicotine vaping on a screener. Additional details on the sample and methods are published elsewhere [18, 19]. Of these 50 youth, 35 participated in the follow-up study. Vaping status did not affect eligibility in the follow-up study, so the sample of 35 included both current and past ENDS users. Participants were ineligible for the study if they did not have personal access to a smartphone. Interview recruitment from the 35 youth ceased when subject saturation was reached [20–23] and included 22 youth. All interviews were phone-based interviews as the study occurred during the COVID-19 shutdown.

### Procedures

Upon Institutional Review Board approval, parent consent and youth assent were obtained for those under 18 years of age, and youth consent was obtained for those 18 and above. One parent did not provide consent for their child to take part in the interview, and three youth declined their participation. Phone interviews were semi-structured and lasted between 30 to 60 min. Each interviewee was also compensated $20 for their time.

### Measures

**Personal characteristics**. The baseline survey gathered basic demographic information about individuals and their families, such as age, sex, and race/ethnicity. Participants reported their socioeconomic status by responding to the following question: “Compared with other people in America, how rich or poor do you consider yourself?”.

**Interview guide**. The in-depth, phone (audio-only) interviews involved questions about vaping behaviors since the pandemic. The interview guide was informed by the socio-ecological framework [14, 15], expertise of the parent project investigators whose knowledge spans vaping and dual-use behaviors among youth, as well as concerns about how the pandemic would impact youth substance use. To learn more about the contexts and motivations associated with youth nicotine and cannabis behaviors during the pandemic, we asked questions that sought to explain the complexities of health risks and vaping (e.g., “How has COVID-19 changed the way you see the benefits of vaping?”). Accordingly, each participant elaborated upon their usual vaping contexts before the pandemic and the extent to which aspects of their nicotine and cannabis use then shifted due to access, availability, and health perceptions.

### Analysis

Interviews were audio recorded after obtaining participants’ verbal consent. Following a transcription protocol [24], interviews were transcribed verbatim and then coded by our team of qualitative data analysts using an applied thematic approach via Dedoose [25, 26]. Two analysts applied deductive codes to segment participants’ narratives into a priori topical categories (e.g., vaping behaviors during COVID-19), drawing on relevant published literature and our previous study findings. Next, analysts identified and applied inductive, content-driven codes for each conceptual category (e.g., how COVID-19 affected nicotine and cannabis vaping behavior) [27, 28]. Inter-coder reliability assessments were conducted during the structural and content coding processes. Two secondary coders also reviewed coding and codebook constructs. The team reviewed and discussed any discrepancies in coding and collaboratively worked to resolve them, achieving clarity and consistency. Codebooks were developed with defined constructs and content for each theme, including sample excerpts and counts, and were revised through an iterative honing process.

## Results

The final sample included 22 youth who participated in the telephone interviews. The majority identified as female (63%) and non-Hispanic white (91%). Current use by substance indicated that 68% vaped nicotine and 55% used cannabis when vaping nicotine in the past 30 days. Additional descriptive statistics are summarized in Table 1.

**Table 1:** Sample characteristics (*N* = 22)

	*N*	%
**Age**		
< 18 years old	6	27.3
≥ 18 years old	16	72.7
**Gender**		
Female	14	63.6
**Ethno-racial identity**^**a**^		
White, Non-Hispanic	20	90.9
Black or African American, Non-Hispanic	3	13.6
**Current education**		
High school	5	22.7
College or vocational school	11	50.0
Not enrolled^b^	6	27.3
**Geographical location**		
Rural area	1	4.5
Small city or town	7	31.8
Suburb near a large city	10	45.5
Large city	4	18.2
**Socioeconomic status**		
Poor	2	9.1
Below average	2	9.1
A little below average	2	9.1
About average	3	13.6
A little above average	6	27.3
Above average	6	27.3
Rich	1	4.5
**Vaping nicotine, past year**^**c**^		
Yes	20	90.9
No	1	4.5
**Vaping nicotine with vaping or smoking cannabis, past year**^**c**^		
Yes	16	72.7
No	5	22.7
**Vaping nicotine, past 30 days (pandemic period)**^**c**^		
Yes	15	68.2
No	5	22.7
**Vaping nicotine with vaping or smoking cannabis, past 30 days (pandemic period)**^**c**^		
Yes	12	54.5
No	4	18.2

Note. Sex assigned at birth and current identification responses did not vary across participants

^a^Participants were able to select more than one race

^b^Indicates high school graduate who is not currently enrolled in postsecondary education

^c^Total may not add up to 22 or 100% due to missing data

Three key domains emerged from our analyses: (1) pandemic-driven changes to vaping frequency and access, (2) reduced social interactions shaping substance- and product-specific use, and (3) motivations and outcomes of dual use (concurrent use or use of two products within two hours of each other) of nicotine and cannabis. Each domain conveys different aspects of vaping that preceded and coincided with the first year of the pandemic.

### Pandemic-driven changes in vaping frequency and access

#### Pandemic as an opportunity to vape less or quit vaping

The majority of participants reported modifying the frequency of their ENDS use since the start of the pandemic. Among them, most participants affirmed that their vaping had decreased, with several noting conscious efforts to quit. Nearly half of the sample disclosed that they had successfully quit prior to the pandemic or were currently attempting to quit and noted that cessation seemed more plausible during the pandemic shutdown.

*“It’s made me look at my habits more and realized I kind of wanted to lay off with it. And I’ve tried quitting more since I’ve been in quarantine.”* [male, 19]

*“I tried to make myself wean down onto different lower strengths of nicotine. And it didn’t work out.”* [female, 18]

Participants motivated to quit took advantage of this temporary suspension from daily life activities, though some were not able to stop vaping or feared that this change could be undone in the future.

“*I just know it’s not good for me. Hopefully, when I go back to school, I won’t do it. But since I’m around people who, like all my friends, who do it, that’s more difficult to not but hopefully, it won’t happen.”* [female, 19]

#### Personal adjustments to peaking health concerns and mandates

Circumstances prompting recent behavior changes were also largely attributed to the seriousness and severity of COVID-19. Often participants took voluntary precautions and acclimated to the enforcement of social distancing safety protocols. To prevent getting sick or getting others sick, for instance, participants sought to purposefully reduce use.

*“I kind of stopped just because I know… It’ll make you more contagious, to be able to catch it.”* [female, 17]

*“I don’t* [vape] *at all. I already have asthma and then, COVID… It just isn’t good for me.”* [female, 18]

*“I won’t hardly do it in public at all, or if I’m around anybody. I know how much that there’s a visual aspect to how much the air that has been inside me spreads.”* [male, 19]

As restrictions on public gatherings instilled concern about ENDS use in outdoor spaces within their communities, participants additionally described the difficulties associated with business closures and access.

*“It’s made it a little bit harder to get things, because all the stores were either closed and what not.”* [female 18]

#### Navigating past and current contexts of use

For some, vaping behaviors stayed the same or increased during the pandemic.

*“Just before COVID, I just wasn’t really vaping. I’m almost doing it more than before.”* [female, 18]

*“I’m guessing probably like, at least once a week, I would be with somebody that’s buying them* [ENDS] *or having me buy.”* [female, 19]

Like the participant above who was still vaping due to being around people buying ENDS and approached to buy for others, several participants acknowledged that while they initially encountered barriers to access (e.g., moving back home from college during the pandemic shutdown), they soon found ways to continue vaping in different environments. Solutions involved finding new people, including family members and older friends, willing to purchase vaping materials (e.g., pens, coils) on their behalf, as well as going to new locations to shop for themselves. Others were able to resume their usual routines as vape stores and gas stations shortly reopened.

*“It* [accessing nicotine during the pandemic] *was probably easier because I’ve come into contact with more people who are 21, but it’s still definitely a struggle but I would say it is easier. My job has opened me up to new relationships.”* [male, 19]

*“I was pretty stressed out that they were closed… But then you had to go in there one at a time and just had to wear a mask.”* [male, 18]

“*Well, it was harder because the vape shops aren't considered as an essential business, or they weren't at first. So, it was hard. Yeah, it was impossible to go to them until they opened. Which was me sort of, like scouring to find, like leftover juice that I had.”* [female, 18]

### Reduced social interactions shaping substance-specific use

#### Interruptions to social environments impacting nicotine use

Participants tended to differ in their use patterns if they owned a personal ENDS versus depending on others in social situations pre-pandemic. For the latter, being unable to convene with these friends resulted in less nicotine vaping.

*“I’m not around other people as much. It’s not as much of a thing for me anymore.”* [female, 19]

*“It’s been harder to get everything. Well, not everything, but harder to get to the things that I need, because of quarantine and shit. No one’s really willing to come outside.”* [male, 18]

Importantly, in the interest of protecting their health, participants were reluctant to use an ENDS (e.g., vaping pen) that did not belong to them. For some participants, prior vaping habits were attributed to social interactions that were now absent.

*“Usually, I would just smack other people’s. But now, it is like, I don’t know whose mouth has been on this!”* [female, 16]

*“I usually only vape when I’m with my friends… And I don’t see them often.”* [female, 18]

#### Intrapersonal factors of vaping and using cannabis

Conversely, such social and relational aspects of nicotine-vaping were not as strongly associated to vaping and smoking cannabis. Rather, participants emphasized personal reasons for using cannabis during the suggested quarantining period that are further contextualized in the next analytic domain.

*“Marijuana, there’s not a lot to do,* [and it] *makes things more fun. It makes more mundane things more fun. Just to kind of enjoy myself a bit more.”* [male, 18]

*“Since I’m home all the time, and that’s kind of unbearable and there’s nothing to do.”* [female, 18]

*“I feel like I’ve had less responsibility since quarantine. I guess loosening up a bit and not being as disciplined, so I’ll let myself smoke* [vape cannabis]*. It’s just something to do if I’m bored or if I want to have a different perception on things going on around me.”* [female, 19]

### Motivations for concurrent use of nicotine and cannabis

#### Dual use as an outlet for well-being and enjoyment

Using nicotine and cannabis together during the pandemic tended to be driven by desires to feel the psychopharmacological effects of simultaneous use. Across participants with consistent or occasional vape patterns as well as those who primarily use either nicotine or cannabis, responses converged to show pleasure-seeking as the foremost determinant of concurrent use.

*“Nicotine gives you a buzz, and then weed gets you high.”* [female, 17]

*“I just think it’s more of an escape, one gives you one… Nicotine will give you one thing and then marijuana will give you another thing. Combined, I feel like combining those two together is like euphoria… If that makes any sense.”* [female, 17]

Such strategic intentions broadly transpired out of the increased time spent at home as recommended by state and local guidelines. While several participants spoke of feelings of gratification and relief obtained from concurrent use, individuals diverged in their reasoning for pursuing this type of use behavior. Some youth laid out the negative impacts of COVID-19 on their mental well-being, namely stress and anxiety. Others, however, acknowledged a newfound boredom.

*“I just feel like it's an escape pretty much for a little bit of what's going on right now because I know not a lot of people want to deal with what's going on right now because that's just a lot to deal with. And also, like, that's, it's helped. I feel like it's helped so many people during this time.”* [female, 17]

*“Because I have more time on my hands, because we close work a lot earlier, because we're not allowed to have anybody inside and stuff like that. So, I guess I just come home have a little more time… I don't really think it's because I'm stressed out or anything extra.”* [male, 18]

#### Unstructured days and less responsibilities

For participants who opened up about their nicotine dependency, the increased amount of idle time associated with stay-at-home orders afforded opportunities to satisfy their cravings. In many instances, youth suggested that they not only vaped more habitually but unreservedly used cannabis concurrently (i.e., around the same time) due to a lack of responsibilities during the pandemic shutdown.

*“In a regular week, senior year… There would always be those five days and that eight-hour block or whatever, where I wouldn’t be doing it, but since you know, at home, it’s a lot easier to just find yourself doing it more because you’re not restricted by that.”* [male, 18]

*“I don't have any tests happening, all of my smoking* [vaping cannabis] *and vaping* [nicotine] *always goes down in the school year.”* [female, 18]

Owing to the added high and buzz derived from concurrent use, a small group of participants that regularly used cannabis expressed vaping with flavored nicotine creates an enhanced experience.

*“I think I’ve picked up vaping* [nicotine] *more as kind of a thing to do when I’m high, because I really don’t vape* [nicotine] *unless I’m high. …I can pick this up and it tastes good. It makes me feel kind of buzzed while I’m also high… Once the pandemic began, I really started to start getting high almost every day.*

*It’s* [vaping flavored nicotine with cannabis] *also kind of like, a different buzz in the middle of a high. It’s a weird feeling but it’s nice.”* [male, 19]

## Discussion

This qualitative study is an essential exploration of youth nicotine and cannabis vaping behaviors during the first year of the COVID-19 pandemic, and the extent to which these practices were modified to adapt to new contexts. Vaping remains a common mode of nicotine and cannabis use yet pandemic-related studies on polysubstance use with cannabis has been limited [13, 29]. Our research offers a nuanced perspective to the novel vaping environment for youth who vape nicotine and use cannabis. Results revealed that profound changes to daily life resulting from the pandemic shaped nicotine and cannabis vaping practices through the following: (1) pandemic-driven changes in vaping frequency and access, (2) reduced social interactions shaping product-specific use, and (3) motivations for and outcomes of concurrent use of nicotine and cannabis.

Consistent with previous studies of young adult substance use behaviors in the first year of the pandemic [10, 13, 30–34], our study showed that youth vaping behaviors varied during the shutdown, with some youth reporting increases in use and others reporting decreases or no significant changes. Regardless of the direction of change, youth identified the uprooting of normal social activities and relationships due to social distancing, closures of non-essential businesses and schools, and quarantining as key factors underlying their use behaviors. In contrast to a recent study linking reduced e-cigarette use to limited access to retail outlets among surveyed young adults [31], our findings paint a more nuanced picture of youth vaping. Indeed, interruptions to nicotine access were encountered, but youth found this to be a temporary challenge that was resolved once their usual store reopened or once they found new retail sites to purchase from, which is consistent with a prior study on vape shops [35]. In contrast to nicotine access, participants’ access to cannabis was not as affected since the informal market, a primary source in Kentucky where laws prohibit the sale of cannabis, remained uninterrupted. In general, youth considered ease of access across substances to be roughly the same as pre-pandemic levels.

Amid assumptions that the pandemic could offer unintended benefits through restricted access and thereby, lower substance use in younger populations [36], our qualitative data expands the narrative on reasons for reduced ENDS use during the pandemic to consider that the lack of in-person socialization and peer interactions, coupled with perceived health risks of sharing ENDS stemming from the pandemic, also resulted in decreased use. The irregular amount of time spent away from friends, school, and social gatherings offered a chance for several participants to implement desired decreases in their nicotine use. Strikingly, the loosening of health precautions (e.g., distanced socialization) included fears that returning to their “normal” lives could prompt a resurgence of social and shared use that would undo their nicotine cessation and quit attempts.

Furthermore, health guidelines encouraging self-isolation dramatically shifted former contexts of use. As with prior studies, youth in our study frequently characterized nicotine vaping as a social activity [32]. Since they were not hanging out with their friends during the pandemic, it was much harder for youth who lacked a personal ENDS to vape. Among them, some also stated concerns about contracting COVID-19 from others and were newly unwilling to engage in this kind of shared use, which corresponds to findings observed in another qualitative study, although its sample consisted of young adults and focused on cannabis vaping [13].

In contrast to nicotine, social isolation contributed to *increases* in vaping and smoking cannabis among participants. Intrapersonal factors were cited, such as managing fatigue and boredom, wanting to detach from their current reality, and needing to alleviate stress or anxiety, all of which overlap with the extant literature on the therapeutic appeal of cannabis [13, 37–39]. Spending more time at home during the early stages of the pandemic being closely associated with more cannabis use may then suggest that cannabis provides a powerful sensation of relief or enjoyment as youth attempt to make sense of the unknown and their life within the context of the pandemic. Concurrent vaping of nicotine and cannabis were similarly linked to pleasure-seeking, yet this framing of use by participants distinguishes it from cannabis-only use during this period. Elevated feelings of euphoria and escape via simultaneous use may indicate that a special yet temporary state of being provides release and relief from pandemic-related stress and anxiety.

Overall, it was evident that differences in motives and rationale for use were identified between and among nicotine and cannabis, and further research is warranted to explore sense-making unique to young populations whose vaping behaviors are strongly connected to social contexts. The complexities of risk embedded in social contexts were prominently illustrated with participants who were afraid that going back to college or seeing friends more regularly again would provoke use and negatively impact their cessation practices and goals. Attention to similar characteristics of the social environment salient to sensemaking can leverage new insights beyond the pandemic for supporting youth wishing to reduce and quit vaping.

Moreover, our work demonstrated that device type played an important role in these health-risk conscious circumstances. Possession of ENDS used for cannabis (i.e., THC) was not unusual, and the availability of cannabis was noted to be more consistent than nicotine. As mentioned, participants that had access to cannabis before the onset of the pandemic had access to cannabis during the pandemic and were, thus, able to continue vaping and smoking with relatively fewer access problems. These findings complement and build upon a qualitative study on nicotine use that reported a host of modifications (i.e., buying less flavored products) that were adopted by young adults to sustain or increase their frequency of tobacco use [12]. Investigating personal ENDS and other modes of cannabis use may help to contextualize observed rises in cannabis-vaping among youth in the early phases of the pandemic [13, 33].

### Limitations and strengths

Despite the exploratory nature of the study, the identified variations in vaping practices highlight how youth are responding in different ways to the seemingly objective presence of a pandemic. Our Kentucky-based sample is not representative of all youth who vape but may call attention to regional characteristics of a tobacco-growing state with high rates of youth vaping. Further, interviews took place in August 2020; hence, the participant experiences and themes that emerged from this study reflect the earlier stages of the COVID-19 pandemic, which are subject to change over time. Also, participants were regularly vaping two years prior to the pandemic yet their experiences and behaviors diverged during the early stages of COVID-19, indicating that, even with such likenesses, sensemaking is multifaceted. The differing narratives may, thereby, be better understood through this theoretical lens that underscores how individuals construct meaning during serious and unpredictable times [16]. We also acknowledge that the interviews captured what participants were willing to divulge, which may be subject to bias and potentially overlook other perceptions and behaviors central to youth who vaped nicotine and/or cannabis during the first year of the pandemic. Finally, youth in our sample tended to use the term “smoke” to refer to vaping and smoking cannabis. Steps were taken to minimize such ambiguities by repeatedly asking youth follow-up questions that specified both the substance of interest and the mode of delivery. Still, the interchangeable use of “vape” and “smoke” to refer to vaping cannabis were not always confirmed. In these few instances, we reference cannabis use generally.

## Conclusion

Despite these constraints, our findings can inform future research that investigates nicotine vaping and cannabis use as a complex phenomenon among youth who vape in different ways within the context of the COVID-19 pandemic and whether such adaptations continue. Larger-scale qualitative studies can be particularly advantageous for prevention and intervention efforts by disentangling how youth who vaped nicotine pre-pandemic are coping with the prolonged presence of COVID-19, as seen with some participants whose use shifted towards cannabis. We recommend that research attend to county-level directives (e.g., lifting mask mandates), individual compliance to such directives, and demographic traits as additional context characteristics of vaping that are relevant to youth and likely to vary over time as the risks of COVID-19 also vary. Studies are needed to also consider how these factors inform vaping and dual-use practices henceforth as well as the sensemaking that accompanies subsequent transitions (e.g., vaccinations). It is imperative to enrich scientific understanding of how each stage of the pandemic has created and can create novel social and personal environments that shape patterns of vaping nicotine and cannabis use and the underlying influences as youth get older and progress into a post-COVID-19 life.

## Data Availability

The data supporting this article are available from the senior author (MA) upon reasonable request.

## References

[1] Czeisler MÉ (2021). Early public adherence with and support for stay-at-home COVID-19 mitigation strategies despite adverse life impact: a transnational cross-sectional survey study in the United States and Australia. BMC Public Health.

[2] Gravely S (2021). Smokers’ cognitive and behavioural reactions during the early phase of the COVID-19 pandemic: findings from the 2020 ITC four country smoking and vaping survey. PLoS ONE.

[3] Usher K, Durkin J, Bhullar N (2020). The COVID-19 pandemic and mental health impacts. Int J Ment Health Nurs.

[4] Medicine TLR (2020). The EVALI outbreak and vaping in the COVID-19 era. Lancet Respir Med.

[5] Chen DT-H, Kyriakos CN (2021). Cigarette and E-Cigarettes Dual Users, Exclusive Users and COVID-19: Findings from Four UK Birth Cohort Studies. Int J Environ Res Public Health.

[6] Gaiha S.M., Cheng J., Halpern-Felsher B. “Association between youth smoking, electronic cigarette use, and COVID-19,.” J Adolesc Health Off Publ Soc Adolesc Med. 2020;67(4):519–23. 10.1016/j.jadohealth.2020.07.002. 10.1016/j.jadohealth.2020.07.002 PMC7417895 32798097

[7] Borgonhi EM, Volpatto VL, Ornell F, Rabelo-da-Ponte FD, Kessler FHP (2021). Multiple clinical risks for cannabis users during the COVID-19 pandemic. Addict Sci Clin Pract.

[8] National Institute on Drug Abuse (NIDA). Percentage of adolescents reporting drug use decreased significantly in 2021 as the COVID-19 pandemic endured. National Institute on Drug Abuse website. https://nida.nih.gov/newsevents/news-releases/2021/12/percentage-of-adolescents-reporting-drug-use-decreased-significantly-in-2021-asthe-covid-19-pandemic-endured. Accessed 21 Dec 2021.

[9] N. I. on D. A. NIDA, “Monitoring the future,” National institute on drug abuse, Dec. 15, 2021. https://www.drugabuse.gov/drug-topics/trends-statistics/monitoring-future (accessed Dec. 21, 2021).

[10] Gaiha SM, Lempert LK, Halpern-Felsher B (2020). Underage youth and young adult e-cigarette use and access before and during the coronavirus disease 2019 pandemic. JAMA Netw Open.

[11] Miech R, Patrick ME, Keyes K, O’Malley PM, Johnston L (2021). Adolescent drug use before and during U.S. national COVID-19 social distancing policies. Drug Alcohol Depend.

[12] Klein E.G., Koopman Gonzalez S., Pike Moore S., Bohnert E.J., Quisenberry A.J., Trapl E.S. “Pulling your mask down to smoke: qualitative themes from young adults on nicotine use during a pandemic,.” Subst Use Misuse. 2021;56(4):437–41. 10.1080/10826084.2020.1869264. 10.1080/10826084.2020.1869264 PMC8078211 33435783

[13] Case KR, Clendennen SL, Shah J, Tsevat J, Harrell MB (2022). Changes in marijuana and nicotine vaping perceptions and use behaviors among young adults since the COVID-19 pandemic: A qualitative study. Addict Behav Rep.

[14] Bronfenbrenner U (1977). Toward an experimental ecology of human development. Am Psychol.

[15] U.S. national cancer institute, “A socioecological approach to addressing tobacco-related health disparities,” U.S. department of health and human services, national institutes of health, national cancer institute, Bethesda, MD, national cancer institute tobacco control monograph 22., 2017. Accessed: Dec. 13, 2021. [Online]. Available: https://cancercontrol.cancer.gov/brp/tcrb/monographs/monograph-22.

[16] Christianson M.K., Barton M.A. “Sensemaking in the Time of COVID‐19,.” J Manag Stud. 2021;58:572. 10.1111/joms.12658.

[17] Weick KE, Sutcliffe KM, Obstfeld D (2005). Organizing and the process of sensemaking. Organ Sci.

[18] Abadi MH (2021). The impact of flavored ENDS use among adolescents on daily use occasions and number of puffs, and next day intentions and willingness to vape. Addict Behav.

[19] Shamblen SR, Abadi MH, Thompson KT, Lipperman-Kreda S, Grube JW, Richard BO (2022). Daily variation in the patterns and characteristics of adolescent ENDS use. Psychol Addict Behav J Soc Psychol Addict Behav.

[20] Coyne IT (1997). Sampling in qualitative research. Purposeful and theoretical sampling; merging or clear boundaries?. J Adv Nurs.

[21] Fusch P, Ness L (2015). Are we there yet? Data Saturation in qualitative research. Qual Rep.

[22] Guest G, Bunce A, Johnson L (2006). How many interviews are enough?: An experiment with data saturation and variability. Field Methods.

[23] Saunders B (2018). Saturation in qualitative research: exploring its conceptualization and operationalization. Qual Quant.

[24] McLellan E, MacQueen KM, Neidig JL (2003). Beyond the qualitative interview: Data preparation and transcription. Field Methods.

[25] Guest G, MacQueen KM, Namey EE. Applied thematic analysis. SAGE. 2011.

[26] Dedoose, web application for managing, analyzing, and presenting qualitative and mixed method research data. Los Angeles, CA: sociocultural research consultants, LLC, 2021.

[27] Bernard H. R, Bernard H. R. Social research methods: qualitative and quantitative approaches. SAGE, 2013.

[28] Miles M. B, Huberman A. M. Qualitative data analysis: An expanded sourcebook. SAGE, 1994.

[29] Smith DM, Kozlowski L, O’Connor RJ, Hyland A, Collins RL (2021). Reasons for individual and concurrent use of vaped nicotine and cannabis: their similarities, differences, and association with product use. J Cannabis Res.

[30] Chaffee BW, Cheng J, Couch ET, Hoeft KS, Halpern-Felsher B (2021). Adolescents’ substance use and physical activity before and during the COVID-19 pandemic. JAMA Pediatr.

[31] Kreslake JM, Simard BJ, O’Connor KM, Patel M, Vallone DM, Hair EC (2021). E-cigarette use among youths and young adults during the COVID-19 pandemic: United States, 2020. Am J Public Health.

[32] Less E. L, Mady M, Beckman K. J, Kingsbury J. H. “If someone has it, i’m gonna hit it’: Lessons learned from Minnesota teens about vaping,” Health Promot. Pract., p. 15248399211045352, Dec. 2021, https://doi.org/10.1177/15248399211045353. 10.1177/15248399211045353 34852204

[33] Nguyen N., Mathur Gaiha S., Halpern-Felsher B. “Self-reported changes in cannabis vaping among US adolescents and young adults early in the COVID-19 pandemic,.” Prev Med Rep. 2021;24:101654. 10.1016/j.pmedr.2021.101654. 10.1016/j.pmedr.2021.101654 PMC8684016 34976701

[34] Stokes AC (2020). Declines in electronic cigarette use among US youth in the era of COVID-19—a critical opportunity to stop youth vaping in its tracks. JAMA Netw Open.

[35] Berg CJ (2021). Vape shop and consumer activity during COVID-19 non-essential business closures in the USA. Tob Control.

[36] Lundahl LH, Cannoy C (2021). COVID-19 and substance use in adolescents. Pediatr Clin North Am.

[37] Blevins CE, Banes KE, Stephens RS, Walker DD, Roffman RA (2016). Motives for marijuana use among heavy-using high school students: An analysis of structure and utility of the comprehensive marijuana motives questionnaire. Addict Behav.

[38] Lee CM, Neighbors C, Woods BA (2007). Marijuana motives: Young adults’ reasons for using marijuana. Addict Behav.

[39] Osborne GB, Fogel C (2008). Understanding the motivations for recreational marijuana use among adult Canadians. Subst Use Misuse.

